# Insertional Achilles tendinopathy is associated with arthritic changes of the posterior calcaneal cartilage: a retrospective study

**DOI:** 10.1186/s13047-015-0103-8

**Published:** 2015-08-25

**Authors:** Ezequiel Palmanovich, Yael Oshri, Yaron S. Brin, Evgeny Edelshtein, Meir Nyska, Iftach Hetsroni

**Affiliations:** Orthopedics Department, Meir Medical Center, Kfar Saba, Israel; Sackler Faculty of Medicine, Tel Aviv University, Tel Aviv, Israel; Family Physician, Clalit Health Service, Sharon Shomron District, Israel; Pathology Department, Meir Medical Center, Kfar Saba, Israel

**Keywords:** Insertional Achilles tendinopathy, Posterior calcaneal wall, Osteoarthritic changes, Histological evaluation, Functional impairment

## Abstract

**Background:**

To characterize the histological changes within the posterior calcaneal cartilage in patients with insertional Achilles tendinopathy (IAT) and test the relationships between severity of the histological changes and level of functional impairment.

**Methods:**

Sixteen posterior calcaneal wall specimens of patients with IAT who had posterior calcaneal ostectomy were investigated. Hematoxylin-eosin stain, Toluidine-blue stain, Polarized light microscopy, and Masson Trichrome stain were used to characterize histological changes. Changes within the posterior calcaneal wall cartilage were graded according to Osteoarthritis Research Society International (OARSI) criteria. Functional scores were completed at the time of surgery according to the American Orthopedic Foot and Ankle Society (AOFAS) Ankle-Hindfoot score.

**Results:**

Mean age of patients was 48.9 years. Histological findings within the posterior calcaneal wall cartilage specimens were consistent with arthritic changes. OARSI grading indicated Grade 2 changes in one specimen, mean AOFAS score 60; Grade 3 changes in three specimens, AOFAS score 73.7 ± 2.5; Grade 4 changes in four specimens, AOFAS score 44 ± 21.4; Grade 5 changes in eight specimens, AOFAS score 48 ± 9.9. Higher OARSI grades were correlated with lower AOFAS scores (*rho* = −0.65, *p* < 0.01).

**Conclusions:**

Degenerative arthritic changes of the posterior calcaneal wall cartilage characterize patients with IAT and the severity of such changes is directly correlated to the degree of functional impairment.

## Background

Insertional Achilles tendinopathy (IAT) is a chronic degenerative process which often affects young active adults. Etiological factors include anatomical dysmorphysm of the posterior calcaneal tubercle, mechanical overload, inflammatory response, and chronic overuse. In one study, the annual incidence in 39 runners was around 10 % [1]. In another study, the cumulative incidence of Achilles tendinopathy before the age 45 was 42 % in former top level middle and long-distance runners compared to only 3 % in matched non-athletic controls with adjusted odds ratio of 31.2 (95 % confidence interval 13.5–71.8, *p* < 0.001) [2].

Non-operative management includes activity modification, physical therapy with eccentric loads, and injections to the retrocalcaneal bursa [3]. In selected patients, when nonoperative treatment fails, surgery may be indicated. The rationale of current surgical techniques for IAT is based on the theory that repeated friction between the anterior aspect of the tendon and the posterior calcaneal wall is a major factor associated with the development of IAT, and therefore decompression in this area can result in reducing contact pressure and repeated friction, primarily during heel dorsiflexion [4]. This is most popularly achieved by ostectomy of the posterior calcaneal wall, and good clinical outcomes have been described following such procedures [5]. Nevertheless, this rationale of repeated tendon friction and overload as the main reason for the development of IAT has been questioned by other observations which in fact showed a strain shielding effect at the anterior surface of the Achilles tendon during repeated heel dorsiflexion that may actually protect the tendon in this specific area from repeated overload injuries [6]. Moreover, other associated factors have also been described, such as systemic inflammatory conditions (seronegative spondyloarthropathies, gout, sarcoidosis, diffuse idiopatic skeletal hyperostosis) [7, 8] and use of some medications such as steroids, contraceptives and hormone replacement therapy [3, 9]. These observations imply that the evolution of IAT may depend on multiple factors, some of which may not be completely understood.

The Achilles tendon insertion complex is composed of three parts: (1) the entheseal fibrocartilage at the tendon-bone junction; (2) the sesamoid fibrocartilage in the deep surface of the tendon; and (3) the periosteal fibrocartilage which covers the opposing surface of the posterior calcaneal wall [10]. In this regard, the degenerative process in each of these three parts may, in theory, play a role in the evolution of IAT. Until today, histological studies focused on changes within the tendon [11] or on changes within the fibrocartilage as observed in animal models only [12]. To the best of our knowledge, the histology of the posterior calcaneal wall cartilage has never been specifically investigated in patients with IAT. The purpose of this study was therefore to characterize the histological changes of the posterior calcaneal wall cartilage in patients with IAT. We hypothesized that IAT is associated with degenerative arthritic changes of the posterior calcaneal wall cartilage, and that the severity of arthritic histological changes is correlated with the severity of functional impairment.

## Methods

Between 2000 and 2007, 38 patients underwent surgery in our foot and ankle service by several orthopedic surgeons due to IAT after fulfilling the following criteria: (1) Failed nonoperative treatment for at least 6 months (rest, physiotherapy, shock wave therapy, steroid injection), and (2) Ultrasound and MRI showing attritional changes at the tendon insertion with increased signal at the tendon or adjacent calcaneal tubercle on T2 MRI supportive of the pathology [13]. The surgery included resection of the posterior calcaneal wall as previously described [5]. For the purpose of this study we included only patients who have completed the American Orthopedic Foot and Ankle Society (AOFAS) Ankle-Hindfoot score [14] at the time of surgery and who had as well a complete histopathological assessment of the posterior calcaneal wall which was excised during surgery. Only patients who had both tests were included in this study, which were all related to one senior orthopedic surgeon (MN) who was leading the study. Two patients who had previous surgery at the posterior calcaneal insertion (previous posterior calcaneal ostectomy or reattachment of Achilles tendon) were excluded. The study therefore included 16 posterior calcaneal wall specimens of 15 patients who underwent posterior calcaneal ostectomy.

### Histological assessment protocol

Hematoxylin-eosin stain was used to delineate soft tissue and cells [13]. Toluidine-blue stain was used to evaluate the severity of degenerative changes within the cartilage [15]. Polarized light microscopy was used to demonstrate early degenerative changes in the cartilage collagen [13]. Masson trichrome stain was used to confirm the presence of fibrous (collagenous) tissue [16]. Severity of changes within the posterior calcaneal wall cartilage was graded according to the Osteoarthritis Research Society International (OARSI) histological criteria [17]. This grading system is considered adequate for cases of mild osteoarthritis and showed high reliability with relatively limited observer experience needed for proper use [18]. The system uses five basic staining procedures appropriate for macroscopic (Indian ink) and histologic (HE/hematoxylin-eosin) visualization and scoring of cartilage proteoglycan and collagen content (toluidine blue/safranin O, and picrosirius red/Goldner’s trichrome), grading the tissue into severity, where 0 describes normal tissue and 5 describes severe osteoarthritis. Grade 6 indicates the worst disease with severe deformity and end-stage bone remodeling. Functional scores were completed at the time of surgery using the American Orthopedic Foot and Ankle Society (AOFAS) Ankle-Hindfoot score which incorporates measures such as pain, gait, range of motion, alignment, and function into one score, ranging 0–100 points. Higher score indicates better function [14]. The study was approved by our Ethical Committee Institutional Review Board (IRB) and patients signed informed consent.

### Statistics

Descriptive statistics included means, ranges, and standard deviations for demographic variables, AOFAS scores, and histological grade. Spearman’s rank correlation coefficient was used to test the relationships between the AOFAS scores and the OARSI Grade. Level of significance was set at *p* < 0.05. IBM, SPSS-22 statistical package software was used for analysis.

## Results

Mean age of the patients was 48.9 years (range 25 to 68 years) (Table 1). There were 11 men. Although the majority of the group was involved in intensive sports, primarily running, four patients had accompanying inflammatory disease which included seronegative spondyloarthropathy, systemic lupus erithematosus, anthiphospholipid antibody syndrome, and ankylosing spondylitis, and three patients had type 2 diabetes. Prior to surgery, all patients were treated with nonsteroidal anti-inflammatory drugs and physiotherapy. Nine patients had local steroid injections and eight had extra-corporeal shock-wave treatment.

**Table 1:** Patient characteristics

Characteristic	Patients (total *n* = 16)
Sex [M]	11 (69 %)
Age, years [Mean, s.d., range]	48.9 ± 12.3 (25–68)
Duration of symptoms [Mean, s.d.]	2.2 ± 2.1 years
Background	
Diabetes type 2	One (6 %)
Inflammatory disease	Four (25 %)
Intensive sports	Nine (56 %)
Specific treatments	
Steroid injection	Nine (56 %)
Shock waves	Eight (50 %)

*M* males; *s.d*. standard deviation

**Fig. 1:**
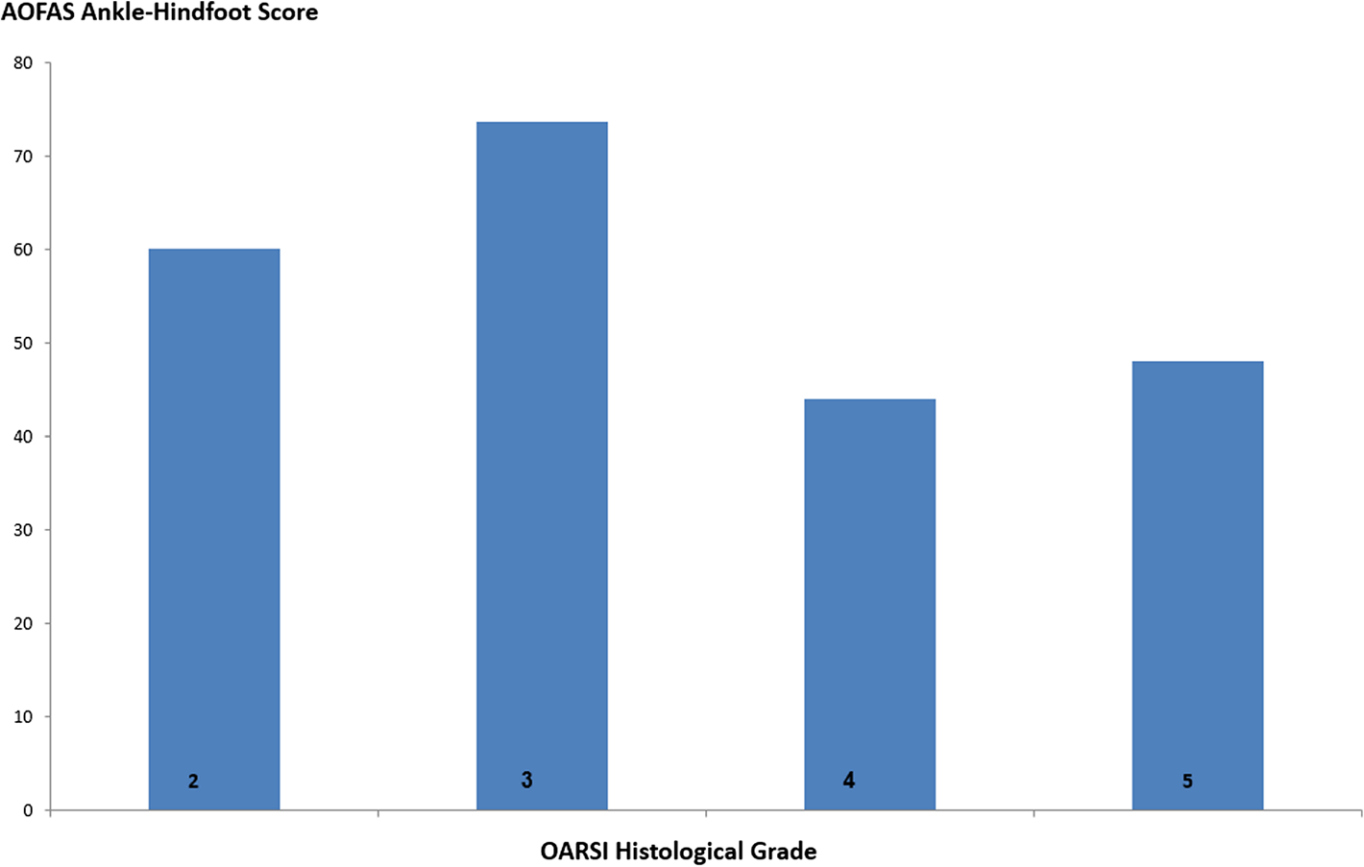
Mean AOFAS Ankle-Hindfoot scores for each OARSI histological grade. OARSI grades were correlated with AOFAS scores (*rho* = −0.65, *p* < 0.01). When OARSI scores are higher (more arthritic changes) the AOFAS score is lower

OARSI grading of the posterior calcaneal wall cartilage indicated grade 2 changes in one specimen, grade 3 changes in three specimens, grade 4 changes in four specimens, and grade 5 changes in eight specimens. The patient who underwent bilateral surgery had the same grade in both operations and had a systemic inflammatory condition. Mean AOFAS score was 60 in the patient with OARSI grade 2; 73.7 ± 2.5 in patients with OARSI grade 3; 44 ± 21.4 in patients with OARSI grade 4; and 48 ± 9.9 in patients with OARSI grade 5 (Fig. 1). Higher OARSI grades were correlated with lower AOFAS scores (*rho* = −0.65, *p* < 0.01).

The following histological findings characterized the specimens.

**Fig. 2:**
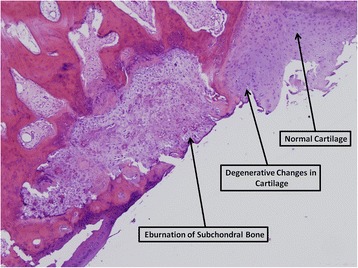
Eburnation of subchondral bone. Bone destruction and formation of tissue deficit. Hematoxilin eosin, 40x

**Fig. 3:**
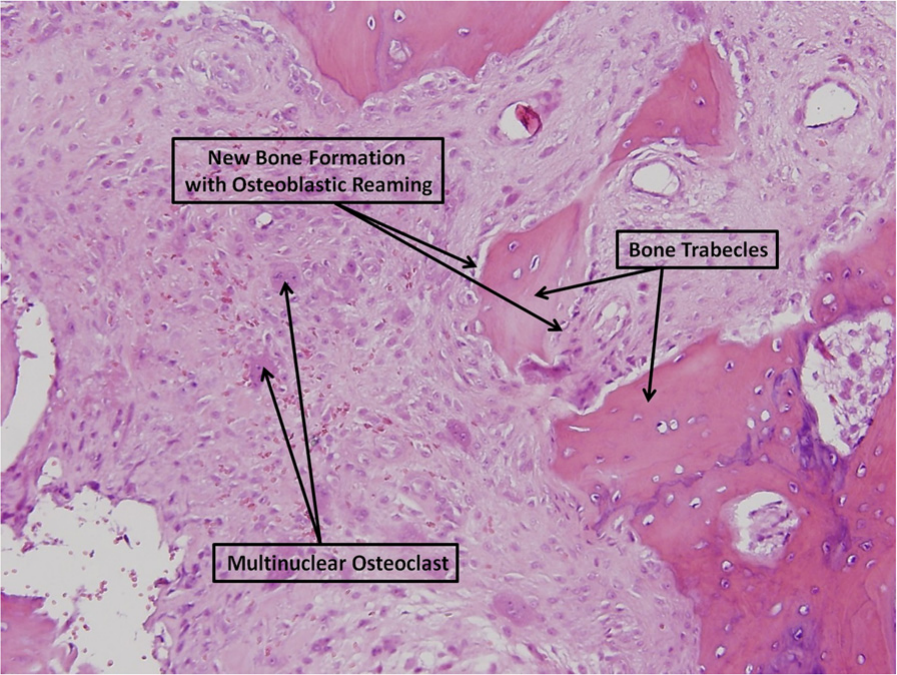
Destruction and construction of new bone. Bone trabecules with osteoblast cover. Osteoclasts scattered in the granulation tissue. Hematoxilin eosin, 100x

**Fig. 4:**
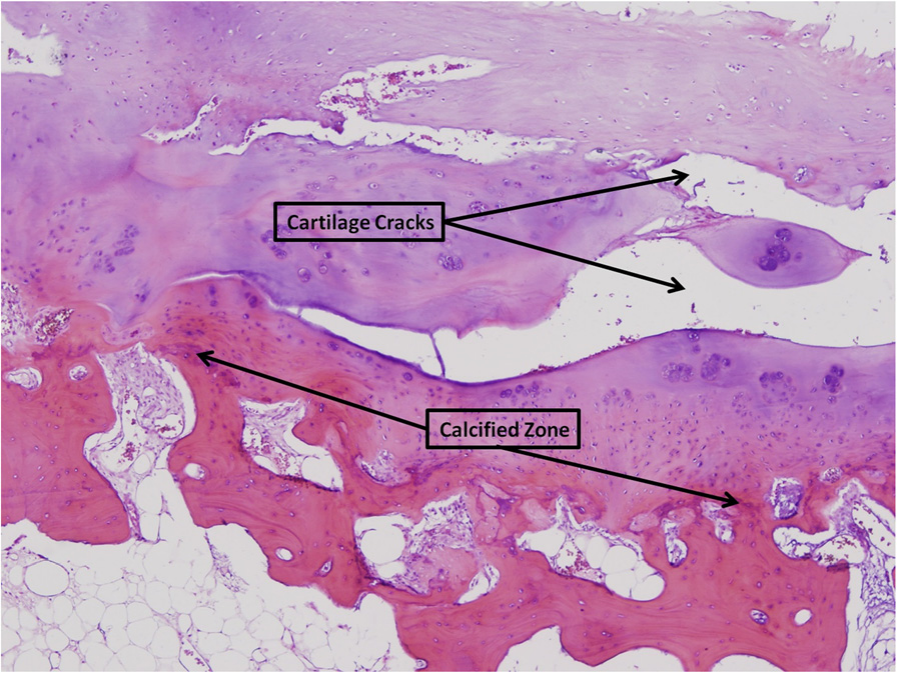
Fibrillation and cracks in the cartilage Hematoxilin eosin, 40x

**Fig. 5:**
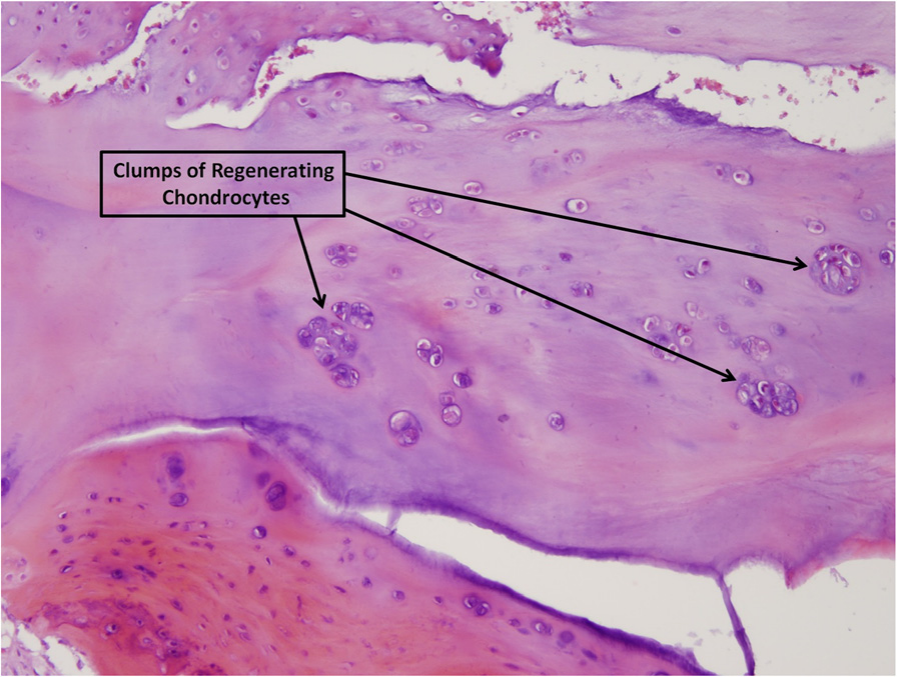
Groups of chondrocytes thrive (healing). Hematoxilin eosin, 100x

**Fig. 6:**
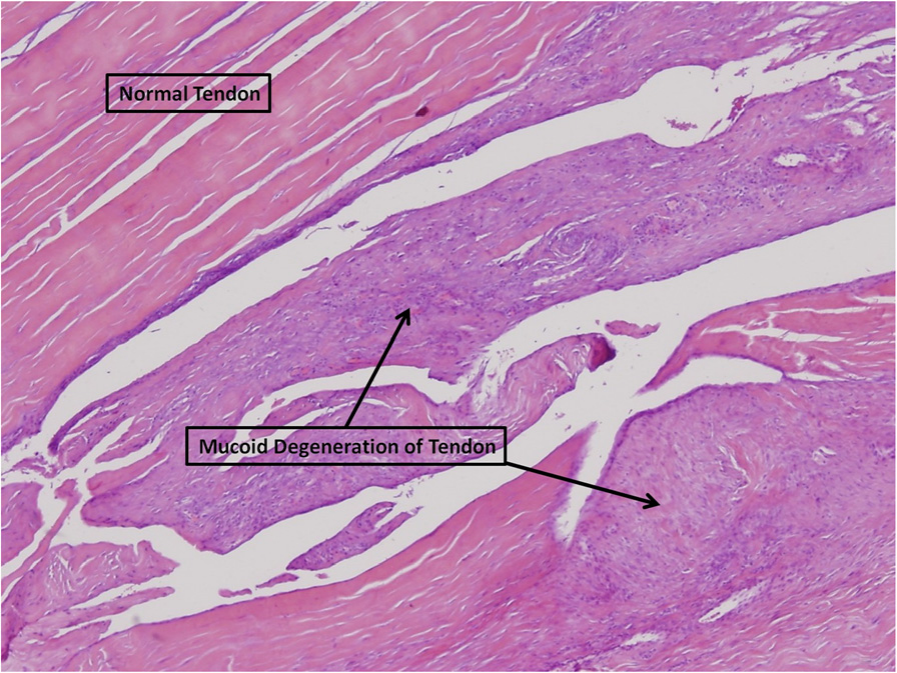
Mucoid changes and cracks in the tendon. Hematoxilin eosin, 40x

- Bone findings: The subchondral bone showed signs of desiccation, destruction of fibroblasts and blood vessels, with osteoclasts, remodeling, fibrous tissue and fat necrosis in the nearby tissue. Bone trabeculae were thin and the bone marrow was fatty and edematous. Cortical bone with new bone formation zones and reaming of osteoblasts and osteoclasts was seen (Figs. 2 and 3).

- Synovium findings: Adjacent to the Achilles tendon, degenerative mucoids were present with proliferation of synovial cells and lymphoplasmocytic perivascular infiltration. Hypertrophy and hyperplasia with papillary formation and numerous blood vessels were observed along with severe edema with follicles and lymphoid infiltrate. Masson histological coloration demonstrated synovial fibrotic changes.

- Cartilage findings: Significant degenerative changes, edema, deep cracks, and fragmentation into calcified zone were observed (Fig. 4). Fibrinoid changes with loose bodies were seen. Masson staining demonstrated irregularities of the collagen fibers with eburnation of the cartilage to subchondral bone. Regenerative changes were found with chondrocytes in the area (Fig. 5).

- Tendon findings: In a minority of samples, scattered mucoid degenerative changes with multiple zones of filtrated blood vessels and lymphocitary infiltrate around the nearby fat tissue were observed. We noticed separation of fibers with the formation of cracks in the tendon (Fig. 6). Gross calcifications were found in areas of cartilage tendon transition.

## Discussion

The main finding of this study was that degenerative arthritic changes of the posterior calcaneal wall cartilage characterized patients with IAT, and that the severity of such changes was correlated with the degree of functional impairment. This observation was based on standard histological stains and findings typical of cartilage wear and tear which result in arthritic degeneration [19]. These findings included erosions of cartilage, hypertrophic and hyperplastic changes in the chondrocytes, remodeling and thickening of adjacent trabeculae, peripheral osteophytes, osteosclerosis, and subchondral cysts [19]. To the best of our knowledge, this observation has not been previously described in patients with IAT, and it may ultimately contribute to the understanding of the pathogenesis and associated contributing factors involved in IAT.

Patients with IAT who do not respond to non-operative management show good clinical outcomes following posterior calcaneal wall ostectomy [5]. The reason for functional improvement and decrease in pain levels following such a procedure is not completely understood. Simple mechanical decompression of the retrocalcaneal space may provide a partial explanation. However, based on the current findings, it can also be argued that by removing degenerated cartilage and exposing the subchondral bone and bone marrow progenitor cells, new fibrocartilage can form within a few weeks. This fibrocartilage may eventually restore the posterior coverage of the calcaneus in a manner that resembles the process observed after removing degenerated cartilage and releasing bone marrow progenitor cells during microfracture of osteochondritis dissecans or other types of degenerated cartilage surfaces in the ankle and knee joints [20]. In the majority of the specimens, we did not observe any inflammatory changes within the tendon. This may further support the fact that fissuring at the tendon insertion, which may contribute to dysfunction, is the result of a process that begins at the adjacent cartilage, and not at the tendon itself. The correlation found between the severity of arthritic changes in the posterior calcaneal wall cartilage and the severity of functional impairment implies that arthritis-modifying agents (IL-1 inhibitors, hyaluronic acid injections, platelet-rich plasma, glucosamine oral supplements, others) [21–23] may be applicable in the treatment of IAT. The clinical usefulness of such agents for the treatment of IAT should yet be tested in properly randomized prospective clinical trials in the future.

Limitations of this study include the relatively small series, the retrospective design with multiple treatments before surgery, some of which may have had some effect on the histological appearance of the specimens’ cartilage, and lacking a control group. Pooling the analysis of patients with and without systemic inflammatory disease may be another potential limitation. It was not however realistic to perform a powerful subgroup analysis in this relatively small series in this respect, but we concur this potential limitation should be taken into account in larger prospective studies that may be conducted in the future with more strict inclusion criteria.

## Conclusion

In conclusion, degenerative arthritic changes of the posterior calcaneal wall cartilage characterize patients with IAT, and the severity of such changes is correlated with the degree of functional impairment. Arthritis-modifying treatments may thus be beneficial in these cases, in addition to physical therapy and load reduction interventions.
